# 1,2-Bis{bis­[4-(trifluoro­meth­yl)phen­yl]phosphino}ethane

**DOI:** 10.1107/S1600536807068547

**Published:** 2008-01-11

**Authors:** Matthew A. Bork, Aaron M. Krueger, Robin S. Tanke, James G. Brummer

**Affiliations:** a Department of Chemistry, University of Wisconsin – Stevens Point, Stevens Point, WI 54481, USA

## Abstract

Crystals of the title compound, C_30_H_20_F_12_P_2_ or *R*_2_PCH_2_CH_2_P*R*_2_ (*R* = 4-C_6_H_4_CF_3_), were inadvertently prepared while attempting to recrystallize a crude sample of *trans*-Re(Cl)(N_2_)(*R*_2_PCH_2_CH_2_P*R*_2_)_2_ from diethyl ether. The molecule lies on a center of inversion. One of the rings lies approximately in the P—C—C—P plane; the dihedral angle is 174.53°.The other ring is not quite perpendicular; the dihedral angle is 71.1°. The compound is isostructural with the *R* = Ph, 4-C_6_H_4_CH_3_ and 4-C_6_H_4_CH_2_CH_3_ analogues. It is well known that the basicity of phosphines and diphosphines can be altered by changing the electron-donating ability of *R*; however, the structural parameters for the title compound do not significantly differ from those of the aforementioned substituted-phenyl compounds.

## Related literature

For the synthesis of the title compound, see: Chatt *et al.* (1985 ▶). For the crystal structures of similar 1,2-bis­(diphenyl­phosphino)ethane structures, see: Tiekink (2001 ▶); Zeller *et al.* (2003 ▶); Zeller & Hunter (2004 ▶). For related literature, see: Allman & Goel (1982 ▶); Larson (1970 ▶); Nordwig *et al.* (2006 ▶); Streuli (1960 ▶); Tolman (1970 ▶).
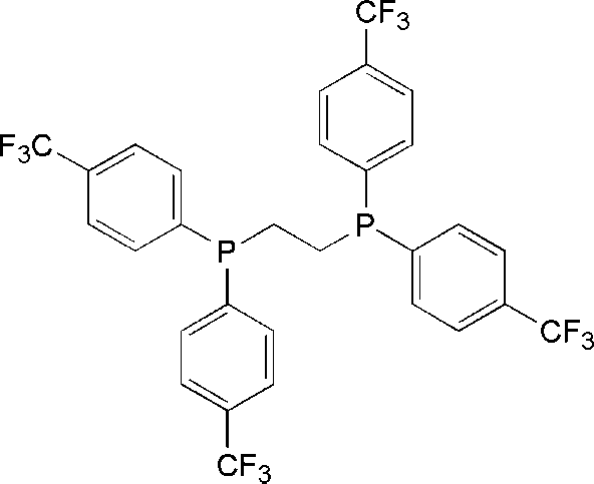


## Experimental

### 

#### Crystal data

C_30_H_20_F_12_P_2_
*M* *_r_* = 670.41
Monoclinic, 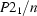

*a* = 15.188 (11) Å
*b* = 5.402 (4) Å
*c* = 18.123 (13) Å
β = 99.044 (9)°
*V* = 1468.3 (19) Å^3^
*Z* = 2
Mo *K*α radiation
μ = 0.24 mm^−1^
*T* = 208 K
0.40 × 0.10 × 0.10 mm

#### Data collection

Bruker SMART APEXII diffractometer
Absorption correction: multi-scan (*SADABS*; Sheldrick, 2006 ▶) *T*_min_ = 0.91, *T*_max_ = 0.98
9947 measured reflections
3240 independent reflections
2616 reflections with *I* > 2σ(*I*)
*R*_int_ = 0.044

#### Refinement

*R*[*F* ^2^ > 2σ(*F* ^2^)] = 0.061
*wR*(*F* ^2^) = 0.162
*S* = 0.95
3229 reflections
208 parameters
H-atom parameters constrained
Δρ_max_ = 0.73 e Å^−3^
Δρ_min_ = −0.43 e Å^−3^

### 

Data collection: *APEX2* (Bruker, 2006 ▶); cell refinement: *APEX2*; data reduction: *APEX2*; program(s) used to solve structure: *SHELXS97* (Sheldrick, 2008 ▶); program(s) used to refine structure: *CRYSTALS* (Betteridge *et al.*, 2003 ▶); molecular graphics: *CAMERON* (Watkin *et al.*, 1996 ▶); software used to prepare material for publication: *CRYSTALS*.

## Supplementary Material

Crystal structure: contains datablocks global, I. DOI: 10.1107/S1600536807068547/om2196sup1.cif

Structure factors: contains datablocks I. DOI: 10.1107/S1600536807068547/om2196Isup2.hkl

Additional supplementary materials:  crystallographic information; 3D view; checkCIF report

## Figures and Tables

**Fig. 1.**
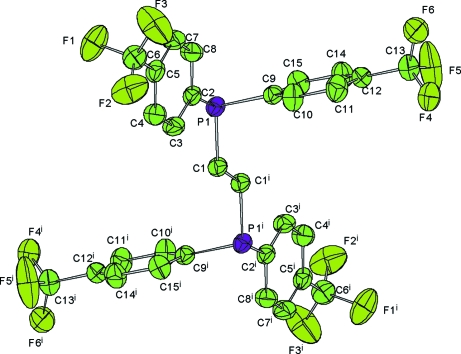
The title compound with displacement ellipsoids drawn at the 50% probability level. Symmetry code: i = -x + 2, -y + 2, -z.

## supplementary crystallographic information

### Comment

1,2-Bis{bis[4-(trifluoromethyl)phenyl]phosphino}ethane was obtained accidently during the recrystallization of *trans*-Re(Cl)(N_2_)(*R*_2_PCH_2_CH_2_P*R*_2_)_2_[*R* = 4-Ph—CF_3_] from diethyl ether. We were interested in preparing this complex in order to measure its luminescent properties and then compare them to those for the analogous *R* = Ph, 4-Ph-OCH_3_, and CH_2_CH_3 _complexes. Our preliminary results indicate that these complexes show simultaneous emission from two excited levels of different orbital parentage. Our intent is to investigate how changes in diphosphine basicity brought about by variations in *R* influence the bandshape and lifetimes of these emissions thereby allowing us to assign the excited states responsible for luminescence.

The title compound resides on a center of inversion. It is isostructural to its *R* = Ph, 4-Ph—CH_3_, and 4-Ph—CH_2_CH_3_ analogues. It is well known that the basicity of phosphines and diphosphines can be altered by changing the electron donating ability of *R*; however, the structural parameters for the title compound do not significantly differ from the aforementioned phenyl substituted compounds.

A summary of the C—P bond distances, C—P—C bond angles, and sums of the C—P—C angles is given in Table 1 for this work and several related diphosphines that contain aromatic and aliphatic substituents. The title compound has nearly identical geoemtric parameters about phosphorus as the other phenyl diphosphines and there appears to be no experimentally significant trends that parallel the electron donating ability of the *para*-substituent, which follows the order CH_3_CH_2_> CH_3_> H > CF_3 _(Nordwig *et al.*, 2006; Allman & Goel, 1982; Tolman, 1970; Streuli, 1960). The aromatic diphosphines display Σ C—P—C values of about 303.5° which indicates a pyramidal arrangement of the bonds about phosphorus. The aliphatic diphosphines are more electron donating with the less sterically demanding *R* = CH_3 _and CH_2_CH_3 _cases giving rise to lower ΣC—P—C values. The *R* = CH(CH_3_)_2_ and C(CH_3_)_3 _compounds display larger ΣC—P—C values and longer C—P bond distances due to increased space requirements for these bulkier substituents. Substituent effects for the alkyl substituted compounds have been discussed previously (Bruckmann & Kruger, 1997; Eisentrager *et al.*, 2003).

One of the rings lines approximately in the P—C—C—P plane; the dihedral angle is 174.53°.The other ring is not quite perpendicular; the dihedral angle is 71.1°.

### Experimental

A non-crystalline sample of *R*_2_PCH_2_CH_2_P*R*_2_ [*R* = 4-Ph—CF_3_] and a crude sample of *trans*-Re(Cl)(N_2_)(*R*_2_PCH_2_CH_2_P*R*_2_)_2_ [*R* = 4-Ph—CF_3_] were prepared according to previously reported methods (Chatt, *et al.*, 1985). Crude *trans*-Re(Cl)(N_2_)(*R*_2_PCH_2_CH_2_P*R*_2_)_2 _was dissolved in a minimum of diethyl ether at 20° C. The yellow-orange solution was filtered and ether was gradually evaporated by passing a slow stream of nitrogen gas through the flask. A mixture of microcrystalline orange solid and pale yellow-orange crystals formed over the course of 4 h. A pale crystal from this mixture was analyzed.

### Refinement

Reflections (11) in the vicinity of the beam stop, with [sin θ/λ] ^2^ < 0.01, were eliminated from the refinement.

All H atoms were positioned geometrically and refined using a riding model, with C—H = 0.93 Å for aromatic H atoms and 0.96 Å for methylene H atoms, and with *U*_iso_(H) = 1.2 *U*_eq_ (C). An extinction correction (Larson, 1970) was applied.

### Crystal data

**Table:**

C_30_H_20_F_12_P_2_	*F*_000_ = 676
*M**_r_* = 670.41	*D*_x_ = 1.516 Mg m^−^^3^
Monoclinic, *P*2_1_/*n*	Melting point: 471 K
Hall symbol: -P 2yn	Mo *K*α radiation λ = 0.71073 Å
*a* = 15.188 (11) Å	Cell parameters from 4592 reflections
*b* = 5.402 (4) Å	θ = 2.3–27.2º
*c* = 18.123 (13) Å	µ = 0.25 mm^−^^1^
β = 99.044 (9)º	*T* = 208 K
*V* = 1468.3 (19) Å^3^	Block, colorless
*Z* = 2	0.40 × 0.10 × 0.10 mm

### Data collection

**Table:**

Bruker SMART APEXII diffractometer	3240 independent reflections
Radiation source: fine-focus sealed tube	2616 reflections with *I* > 2σ(*I*)
Monochromator: graphite	*R*_int_ = 0.044
*T* = 208 K	θ_max_ = 27.2º
ω scans	θ_min_ = 1.6º
Absorption correction: multi-scan(SADABS; Sheldrick, 2006)	*h* = −19→19
*T*_min_ = 0.91, *T*_max_ = 0.98	*k* = −4→6
9947 measured reflections	*l* = −23→17

### Refinement

**Table:**

Refinement on *F*^2^	Hydrogen site location: inferred from neighbouring sites
Least-squares matrix: full	H-atom parameters constrained
*R*[*F*^2^ > 2σ(*F*^2^)] = 0.061	Method = Modified Sheldrick *w* = 1/[σ^2^(*F*^2^) + ( 0.07*P*)^2^ + 1.82*P*] ,where *P* = (max(*F*_o_^2^,0) + 2*F*_c_^2^)/3
*wR*(*F*^2^) = 0.162	(Δ/σ)_max_ = 0.001
*S* = 0.95	Δρ_max_ = 0.73 e Å^−^^3^
3229 reflections	Δρ_min_ = −0.43 e Å^−^^3^
208 parameters	Extinction correction: Larson (1970), Equation 22
Primary atom site location: structure-invariant direct methods	Extinction coefficient: 100 (30)

### Fractional atomic coordinates and isotropic or equivalent isotropic displacement parameters (Å^2^)

**Table:**

	*x*	*y*	*z*	*U*_iso_*/*U*_eq_	
F1	1.27583 (17)	0.8551 (5)	0.42720 (14)	0.0973	
F2	1.22595 (19)	0.5004 (4)	0.40090 (12)	0.0933	
F3	1.15394 (18)	0.7552 (7)	0.45867 (12)	0.1188	
F4	0.58890 (16)	0.7965 (6)	0.11270 (19)	0.1126	
F5	0.56328 (18)	1.1552 (8)	0.0760 (3)	0.1601	
F6	0.59438 (18)	1.0822 (8)	0.19080 (19)	0.1525	
C1	1.02538 (17)	0.9577 (5)	0.03782 (13)	0.0376	
C2	1.06731 (16)	1.0281 (5)	0.19636 (13)	0.0366	
C3	1.12029 (18)	0.8196 (5)	0.19746 (14)	0.0432	
C4	1.16363 (19)	0.7219 (6)	0.26452 (15)	0.0474	
C5	1.15250 (17)	0.8323 (5)	0.33151 (14)	0.0422	
C6	1.1999 (2)	0.7337 (7)	0.40384 (16)	0.0566	
C7	1.0996 (2)	1.0400 (6)	0.33167 (15)	0.0501	
C8	1.05801 (19)	1.1393 (6)	0.26470 (15)	0.0466	
C9	0.89383 (17)	1.1273 (5)	0.11969 (14)	0.0375	
C10	0.8645 (2)	0.9282 (6)	0.15831 (18)	0.0531	
C11	0.7744 (2)	0.8957 (7)	0.16050 (19)	0.0600	
C12	0.71295 (19)	1.0603 (6)	0.12370 (17)	0.0530	
C13	0.6158 (2)	1.0234 (9)	0.1269 (3)	0.0781	
C14	0.7406 (2)	1.2566 (7)	0.08562 (19)	0.0587	
C15	0.83094 (19)	1.2916 (6)	0.08420 (17)	0.0488	
P1	1.01119 (4)	1.18369 (12)	0.11177 (3)	0.0363	
H11	1.0876	0.9429	0.0344	0.0451*	
H12	1.0028	0.7998	0.0503	0.0451*	
H31	1.1270	0.7433	0.1527	0.0513*	
H41	1.2006	0.5845	0.2647	0.0539*	
H71	1.0918	1.1122	0.3768	0.0578*	
H81	1.0232	1.2810	0.2648	0.0543*	
H101	0.9059	0.8154	0.1819	0.0610*	
H111	0.7548	0.7643	0.1868	0.0685*	
H141	0.6989	1.3674	0.0614	0.0680*	
H151	0.8499	1.4260	0.0587	0.0563*	

### Atomic displacement parameters (Å^2^)

**Table:**

	*U*^11^	*U*^22^	*U*^33^	*U*^12^	*U*^13^	*U*^23^
F1	0.0832 (16)	0.114 (2)	0.0773 (15)	−0.0152 (14)	−0.0410 (12)	0.0092 (14)
F2	0.135 (2)	0.0749 (15)	0.0578 (13)	0.0188 (14)	−0.0231 (13)	0.0109 (11)
F3	0.0998 (19)	0.211 (3)	0.0491 (12)	0.057 (2)	0.0224 (12)	0.0483 (17)
F4	0.0612 (14)	0.128 (2)	0.155 (3)	−0.0417 (15)	0.0383 (15)	−0.065 (2)
F5	0.0438 (14)	0.213 (4)	0.219 (4)	0.0033 (19)	0.0043 (19)	0.058 (3)
F6	0.0792 (17)	0.252 (4)	0.143 (3)	−0.069 (2)	0.0692 (18)	−0.128 (3)
C1	0.0375 (12)	0.0447 (14)	0.0301 (12)	−0.0025 (11)	0.0036 (9)	−0.0012 (10)
C2	0.0352 (12)	0.0416 (13)	0.0321 (11)	−0.0035 (10)	0.0022 (9)	−0.0015 (10)
C3	0.0476 (14)	0.0499 (15)	0.0306 (12)	0.0024 (12)	0.0015 (10)	−0.0059 (11)
C4	0.0480 (15)	0.0504 (16)	0.0419 (14)	0.0086 (12)	0.0012 (11)	−0.0001 (12)
C5	0.0368 (13)	0.0543 (16)	0.0338 (12)	−0.0062 (11)	0.0003 (10)	0.0016 (11)
C6	0.0545 (17)	0.076 (2)	0.0371 (14)	0.0052 (16)	−0.0006 (13)	0.0024 (14)
C7	0.0527 (16)	0.0644 (19)	0.0323 (13)	0.0042 (14)	0.0036 (11)	−0.0087 (12)
C8	0.0488 (15)	0.0506 (16)	0.0396 (14)	0.0089 (12)	0.0043 (11)	−0.0060 (12)
C9	0.0380 (12)	0.0408 (13)	0.0330 (12)	−0.0019 (10)	0.0041 (10)	−0.0020 (10)
C10	0.0447 (15)	0.0549 (18)	0.0584 (17)	−0.0033 (13)	0.0041 (13)	0.0165 (14)
C11	0.0525 (17)	0.066 (2)	0.0627 (19)	−0.0154 (15)	0.0142 (15)	0.0084 (16)
C12	0.0403 (14)	0.068 (2)	0.0514 (16)	−0.0054 (14)	0.0108 (12)	−0.0176 (15)
C13	0.0438 (18)	0.106 (3)	0.087 (3)	−0.011 (2)	0.0157 (18)	−0.029 (2)
C14	0.0437 (16)	0.067 (2)	0.0638 (19)	0.0105 (15)	0.0027 (14)	0.0007 (16)
C15	0.0469 (15)	0.0491 (16)	0.0507 (16)	0.0045 (13)	0.0090 (12)	0.0101 (13)
P1	0.0368 (4)	0.0389 (4)	0.0320 (3)	−0.0041 (3)	0.0022 (2)	0.0012 (2)

### Geometric parameters (Å, °)

**Table:**

F1—C6	1.336 (4)	C2—C3	1.383 (4)
F2—C6	1.324 (4)	C2—C8	1.403 (4)
F3—C6	1.307 (4)	C2—P1	1.836 (3)
F4—C13	1.305 (5)	C3—C4	1.392 (4)
F5—C13	1.328 (6)	C3—H31	0.930
F6—C13	1.290 (5)	C4—C5	1.387 (4)
H101—C10	0.930	C5—C6	1.492 (4)
H111—C11	0.930	C5—C7	1.380 (4)
H141—C14	0.930	C7—C8	1.385 (4)
H151—C15	0.930	C9—C10	1.394 (4)
H41—C4	0.930	C9—C15	1.386 (4)
H71—C7	0.930	C9—P1	1.836 (3)
H81—C8	0.930	C10—C11	1.386 (4)
C1—C1^i^	1.533 (5)	C11—C12	1.383 (5)
C1—P1	1.850 (3)	C12—C13	1.499 (5)
C1—H11	0.960	C12—C14	1.367 (5)
C1—H12	0.960	C14—C15	1.389 (4)
			
C1^i^—C1—P1	110.6 (2)	C7—C8—H81	119.9
C1^i^—C1—H11	109.3	C10—C9—C15	118.4 (3)
P1—C1—H11	109.2	C10—C9—P1	123.9 (2)
C1^i^—C1—H12	109.1	C15—C9—P1	117.7 (2)
P1—C1—H12	109.2	C9—C10—H101	119.3
H11—C1—H12	109.5	C9—C10—C11	120.5 (3)
C3—C2—C8	118.3 (2)	H101—C10—C11	120.2
C3—C2—P1	125.28 (19)	C10—C11—H111	120.6
C8—C2—P1	116.3 (2)	C10—C11—C12	120.0 (3)
C2—C3—C4	121.0 (2)	H111—C11—C12	119.4
C2—C3—H31	119.5	C11—C12—C13	119.3 (3)
C4—C3—H31	119.5	C11—C12—C14	120.3 (3)
C3—C4—H41	120.6	C13—C12—C14	120.4 (3)
C3—C4—C5	119.7 (3)	C12—C13—F5	112.9 (4)
H41—C4—C5	119.7	C12—C13—F4	113.4 (3)
C4—C5—C6	120.5 (3)	F5—C13—F4	103.3 (4)
C4—C5—C7	120.2 (2)	C12—C13—F6	113.0 (3)
C6—C5—C7	119.3 (3)	F5—C13—F6	106.4 (4)
C5—C6—F1	112.1 (3)	F4—C13—F6	107.0 (4)
C5—C6—F2	114.2 (3)	H141—C14—C12	119.8
F1—C6—F2	103.4 (3)	H141—C14—C15	120.4
C5—C6—F3	113.2 (3)	C12—C14—C15	119.8 (3)
F1—C6—F3	104.7 (3)	C14—C15—C9	121.0 (3)
F2—C6—F3	108.3 (3)	C14—C15—H151	119.9
C5—C7—H71	119.8	C9—C15—H151	119.1
C5—C7—C8	119.9 (3)	C1—P1—C2	102.20 (13)
H71—C7—C8	120.3	C1—P1—C9	99.93 (12)
C2—C8—C7	120.8 (3)	C2—P1—C9	100.83 (13)
C2—C8—H81	119.3		
			
C2—P1—C1—C1^i^	174.53 (18)	C7—C5—C6—F2	160.7 (3)
C9—P1—C1—C1^i^	71.1 (2)	C4—C5—C6—F3	−146.1 (3)
C2—P1—C9—C10	−25.8 (3)	C7—C5—C6—F1	−82.2 (3)
C1—P1—C2—C3	11.2 (3)	C7—C5—C6—F3	36.0 (4)
C9—P1—C2—C3	113.9 (2)	C5—C7—C8—C2	1.4 (5)
C1—P1—C2—C8	−171.8 (2)	P1—C9—C10—C11	−178.7 (2)
C9—P1—C2—C8	−69.1 (2)	C15—C9—C10—C11	0.4 (4)
C1—P1—C9—C15	−100.4 (2)	P1—C9—C15—C14	177.9 (2)
C1—P1—C9—C10	78.8 (3)	C10—C9—C15—C14	−1.3 (4)
C2—P1—C9—C15	155.1 (2)	C9—C10—C11—C12	0.5 (5)
P1—C1—C1^i^—P1^i^	179.98 (16)	C10—C11—C12—C13	−179.7 (4)
P1—C2—C3—C4	176.8 (2)	C10—C11—C12—C14	−0.6 (5)
C3—C2—C8—C7	−1.1 (4)	C11—C12—C13—F4	−47.9 (5)
C8—C2—C3—C4	−0.1 (4)	C11—C12—C13—F5	−165.0 (4)
P1—C2—C8—C7	−178.4 (2)	C11—C12—C13—F6	74.1 (5)
C2—C3—C4—C5	1.1 (4)	C14—C12—C13—F4	133.0 (4)
C3—C4—C5—C7	−0.8 (4)	C14—C12—C13—F5	15.8 (6)
C3—C4—C5—C6	−178.7 (3)	C14—C12—C13—F6	−105.0 (5)
C4—C5—C6—F1	95.7 (4)	C11—C12—C14—C15	−0.3 (5)
C4—C5—C6—F2	−21.5 (4)	C13—C12—C14—C15	178.8 (3)
C4—C5—C7—C8	−0.4 (4)	C12—C14—C15—C9	1.3 (5)
C6—C5—C7—C8	177.5 (3)		

Symmetry codes: (i) −*x*+2, −*y*+2, −*z*.

### Table 1 Comparison of structural parameters (Å, °) for R_2_PCH_2_CH_2_PR_2_

**Table:**

R	C-P-C	Σ C-P-C	P-C_ethyl_	P-C_R_
p-Ph-CF_3_^a^	100.79, 102.15,99.96	302.90	1.854	1.838
Ph^b^	100.219,102.369,101.047	303.64	1.844	1.832
p-Ph-CH_3_^c^	98.668,101.864,102.985	303.52	1.849	1.821
p-Ph-CH_2_CH_3_^d^	99.719,102.754,101.37	303.84	1.85	1.83
CH_3_^e^	98.869,99.665,98.872	297.3	1.848	1.836
CH_2_CH_3_^e^	99.272,99.491,100.206	298.5	1.845	1.843
CH(CH_3_)_2_^e^	101.184,100.805,102.235	304.2	1.86	1.86
C(CH_3_)_3_^f^	100.990,103.197,110.350	314.5	1.86	1.89

Notes: (a) This work; (b) Tiekink (2001); (c) Zeller *et al.* (2003); (d) Zeller & Hunter (2004); (e) Bruckmann & Kruger (1997); (f) Eisentrager *et al.* (2003).
